# Preliminary injury risk estimation for occupants involved in frontal crashes by combining computer simulations and real crashes

**DOI:** 10.1177/0036850420908750

**Published:** 2020-04-24

**Authors:** Jinlong Qiu, Sen Su, Aowen Duan, Chengjian Feng, Jingru Xie, Kui Li, Zhiyong Yin

**Affiliations:** 1 Chongqing Key Laboratory of Vehicle Crash/Bio-impact and Traffic Safety, Institute of Surgery Research, Third Affiliated Hospital, Army Medical University, Chongqing, China; 2 First Affiliated Hospital, Army Medical University, Chongqing, China; 3 Institute of Surgery Research, Third Affiliated Hospital, Army Medical University, Chongqing, China

**Keywords:** Automotive collision safety, injury prediction, traffic accidents, neural network, occupant injury

## Abstract

The fatality rate can be dramatically reduced with the help of emergency medical services. The purpose of this study was to establish a computational algorithm to predict the injury severity, so as to improve the timeliness, appropriateness, and efficacy of medical care provided. The computer simulations of full-frontal crashes with rigid wall were carried out using LS-DYNA and MADYMO under different collision speeds, airbag deployment time, and seatbelt wearing condition, in which a total of 84 times simulation was conducted. Then an artificial neural network is adopted to construct relevance between head and chest injuries and the injury risk factors; 37 accident cases with Event Data Recorder data and information on occupant injury were collected to validate the model accuracy through receiver operating characteristic analysis. The results showed that delta-v, seatbelt wearing condition, and airbag deployment time were important factors in the occupant’s head and chest injuries. When delta-v increased, the occupant had significantly higher level of severe injury on the head and chest; there is a significant difference of Head Injury Criterion and Combined Thoracic Index whether the occupant wore seatbelt. When the airbag deployment time was less than 20 ms, the severity of head and chest injuries did not significantly vary with the increase of deployment time. However, when the deployment time exceeded 20 ms, the severity of head and chest injuries significantly increased with increase in deployment time. The validation result of the algorithm showed that area under the curve = 0.747, *p* < 0.05, indicating a medium level of accuracy, nearly to previous model. The computer simulation and artificial neural network have a great potential for developing injury risk estimation algorithms suitable for Advanced Automatic Crash Notification applications, which could assist in medical decision-making and medical care.

## Introduction

According to World Health Organization, the number of road traffic deaths continues to climb, reaching a high of 1.35 million in 2016. Road traffic injuries have become the eighth leading cause of death for all age.^1^ Although passive and active safety techniques have been developed for the purpose of occupant injury prevention. traffic accidents still rank as one of the most dominant causes of death and injury globally. After collision, the available of timely medical rescue is associated with reduction of death rates. According to the statistics of traffic accidents, the fatality rate can be reduced by 17 to 29 percent with the help of timely emergency medical services (EMS).^2^

Based on the related result in previous research, almost 76.9% causalities transferred to the emergency medical centers suffered from minor injury,^3^ which has led to an unnecessary burden on emergency medical resources. More specifically, a more timely and accurate assessment of the injury severity and condition of the occupant involved in a vehicle collision could significantly improve the allocation of the emergency medical resources.

Efforts toward the assessment of injury severity sustained by vehicle crash victims have been helped by technologies such as Advanced Automatic Crash Notification (AACN) systems. However, for the commercialization of an AACN model, it is essential to develop an algorithm that can estimate the occupant’s injury severity based on physical metrics associated with collision. The most widely used model is the Urgency Algorithm. While the Urgency Algorithm may predict the overall occurrence of severe occupant injuries (i.e. Abbreviated Injury Scale (AIS) 3+ injury),^4^ the applicability of this algorithm for the EMS is rather limited due to its lack of specificity regarding the injured body region(s) and its inability to detect life-threatening occult injuries.^5^

The Event Data Recorder (EDR), a device implanted in the airbag control module, is used for recording crash data such as the speed, acceleration, delta-v, seatbelt pre-tensioning time, airbag deployment time, and seatbelt wearing condition.^6^ Those metrics may highly relate to the injury severity of the occupant but difficult to access through real-world crash reconstruction. Therefore, the EDR data could be used for modeling and validation of the injury prediction algorithm.^7,8^

Considering the limitation of previous injury predicting algorithms, injury risk prediction method for body region-specific should be established. Head and chest regions are given priority, because they are predominated causes of death in traffic accidents. The objective of this study was to develop a computational methodology to explore the factors influencing the body region-specific (head and chest) injury, then build the injury risk algorithm based on the related parameters using artificial neural network, and validate it with real crash data from the EDRs.

## Materials and methods

### Injury reconstruction

**Figure 1.**
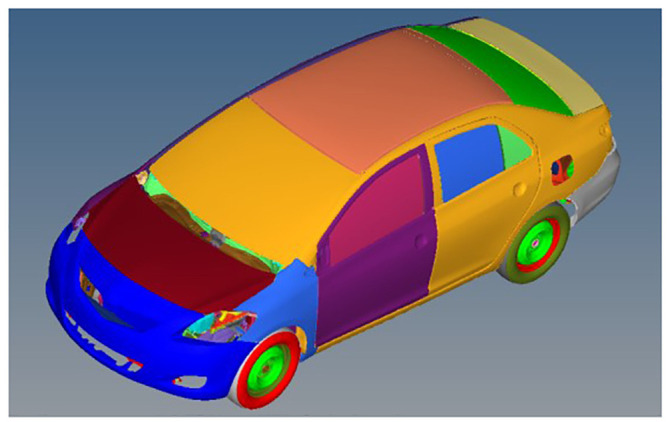
Yaris vehicle FE model.

**Figure 2.**
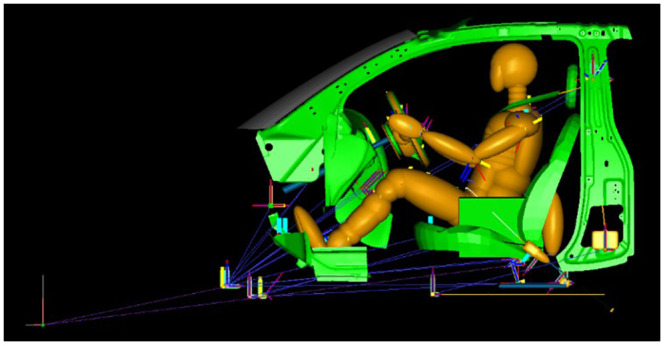
Yaris vehicle-occupant MADYMO model.

Computer simulations of full-frontal crashes with rigid wall were carried out using LS-DYNA under different collision speeds. The Yaris (Toyota) Finite Element (FE) model, validated by National Crash Analysis Center (NCAC), was adopted,^9^ which contains 970,000 meshes and 771 parts (see Figure 1). The multi-rigid body model contains vehicle, also NCAC-verified Yaris model and a Hybrid III 50th Percentile Male (see Figure 2).

The vehicle’s longitudinal acceleration calculated from the FE simulation model, as a boundary condition, was uploaded to the multi-rigid model using dynamics analysis software, MADYMO. The values outputted from MADYMO were used to estimate the injuries under varied conditions of collision speed, airbag deployment time, and seatbelt conditions. The collision speeds were set at six levels, that is, 30, 40, 50, 56, 70, and 80 km/h. The airbag deployment time was set at seven levels, that is, 2, 5, 10, 20, 30, 40, and 50 ms. The occupants were belted or unbelted. The mixed-level orthogonal experimental designs were performed for the simulations under different parameters and levels, in which a total of 84 simulations were conducted.

Head Injury Criterion (HIC) was evolved from the Wayne State Tolerance Curve (WSTC) to relate cadaver impacts to brain injury.^10^ HIC has been proposed by National Highway Traffic Safety Administration (NHTSA) as an effective tool to assess head injury and is computed according to the following expression

(1) $$\mathrm{HIC}=\max{\left[\frac{1}{t_{2}-t_{1}}\int_{t_{1}}^{t_{2}}a\left(t\right)\mathrm{dt}\right]}^{2.5}\left(t_{2}-t_{1}\right)$$

where *t*_2_ and *t*_1_ are any two arbitrary times during the acceleration pulse. NHTSA proposed to limit this HIC time interval to 36 ms. Now HIC_36_ has been widely used to assessing head injury in automotive safety industry.^11,12^

Many researchers provided a measure of prediction of chest injury outcome, a formulation that included both peak chest acceleration and maximum chest deflection, called the Combined Thoracic Index (CTI). CTI appeared to provide superior predictive capability compared to one single variable, such as peak chest acceleration, peak chest deflection, or the Viscous Criterion (*V* × *C*).^13,14^ The formulation of the CTI is

(2) $$\mathrm{CTI}=\frac{A_{\max}}{A_{\mathrm{int}}}+\frac{D_{\max}}{D_{\mathrm{int}}}$$

where *A*_max_ and *D*_max_ are the maximum observed acceleration and deflection, and *A*_int_ and *D*_int_ are the corresponding maximum allowable intercept values.

Therefore, HIC_36_ and CTI calculated by computer simulation were used as head and chest injury criterion. However, these two criteria were commonly used in computer simulation or injury evaluation of crash dummy, which cannot be obtained in real crash. In real-world accidents, the injury severity is described using AIS codes. The AIS is a dictionary specifically designed as a system to define the severity of injuries throughout the body, such as fracture, hemorrhage, and soft tissue injury. In addition to a universal injury language, it provides measures of injury severity that can be used to stratify and classify injury severity in all body regions.^15^

To provide probabilistic estimates of region-specific injury risk as a function of their severities, NHTSA established the injury risk functions between injury metrics (e.g. HIC, *N_ij_*, CTI) and the AIS code.^16,17^ The injury risk functions are described in Appendix 1, in order to determine the range of HIC/CTI values in one certain AIS code. Other researchers usually take assuming equal severity ratios between corresponding risk curves for HIC/CTI and AIS at 50% risks.^18^ The same hypothesis was used in this study. The established relevance of HIC/CTI with AIS code is as listed in Table 1.

**Table 1.** The relevance of HIC and CTI with AIS code.

Injury severity	AIS	HIC	CTI
None or minor injury	0–1	<250	<0.80
Moderate injury	2	<750	<1.15
Major injury	3	<1250	<1.39
Acute injury	4	<1750	<2.16
Critical death	5–6	>1750	>2.16

HIC: Head Injury Criterion; CTI: Combined Thoracic Index; AIS: Abbreviated Injury Scale.

### Injury prediction model

The values of HIC and CTI, outputted from the simulation under different initial parameters, were analyzed to select the factors influencing head and chest injuries. An artificial neural network was then adopted to construct the relevance between head as well as chest injuries and the injury factors. In this process, the injury factor values were input, and the AIS codes for the occupant’s head and chest injuries were the output values for constructing three-layered feedforward neural networks. The maximum training times were set as 10,000, and the learning rate at 0.3. After training, the neural network produced non-linear mapping relation between the injury factors and the severity of occupant’s head and chest injuries when the accuracy reaches to 0.01

(3) $$\left[H_{\mathrm{AIS}},C_{\mathrm{AIS}}\right]=f\left(I_{1}+I_{2}+I_{3}+I_{4}\cdots \right)$$

In this equation, *H_AIS_, C_AIS_, I*, and *f* represent severity of head injury, severity of chest injury, injury factor, and non-linear mapping relation, respectively.

### Model validation

In total, 37 accident cases with EDR data and information on occupant injury were collected through in-depth investigation of traffic accidents. The data source was the Chongqing Bayi Traffic Accident Appraisal Centre in China. The selected cases included only frontal collision with colliding angles between negative (–) 45° and positive (+) 45°, and injuries to the driver position victims were only considered. Meanwhile, cases involved with rollover or multiple collisions were excluded. The injury-related parameters recorded in EDRs were inputted into the neural network model for the calculation of predicted values of head and chest injuries. The predicted injuries were then compared with the actual injuries for validation of the model’s accuracy.

The accuracy of the model was validated with receiver operating characteristic (“ROC”) analysis, which has been widely used in illustrating and evaluating the diagnostic or predictive performance.^19^ The severity of the injury was categorized into two levels: when Maximum Abbreviated Injury Scale (MAIS) was greater than or equal to 3, the injury was classified as a serious injury, and when MAIS was less than 3 it was classified as a non-serious injury. Table 2 summarizes the classification matrix for ROC analysis. The measures A and D are related to cases with accurate injury prediction by the present algorithm. The measure B relates to cases predicted to be a non-serious injury but was actually a serious. On the contrary, the measure C comprises of cases predicted to be a serious injury but was actually a non-serious injury. In a ROC analysis graph, the vertical axis represents the rate of predicted serious injury to actual serious injury. The horizontal axis represents 1 minus the rate of “predicted non-serious injury” accidents to “actual non-serious injury” accidents. The accuracy of the algorithm is represented by the area under the curve (“AUC”). When AUC is greater than 0.9, it means the algorithm have a high accuracy; when AUC is between 0.7 and 0.9, it means medium-level accuracy; and when AUC is between 0.5 and 0.7, it means low accuracy.^19^ The ROC analysis was conducted by SPSS 18.0 software, and *p* < 0.05 was considered to be statistically significant.

**Table 2.** Classification matrix for ROC analysis.

Prediction of injury	Results of injury	
	Serious injury	Non-serious injury
Serious injury	A TP: True Positive	C FP: False Positive
Non-serious injury	B FN: False Negative	D TN: True Negative

ROC: receiver operating characteristic.

## Results

### Simulation results

**Figure 3.**
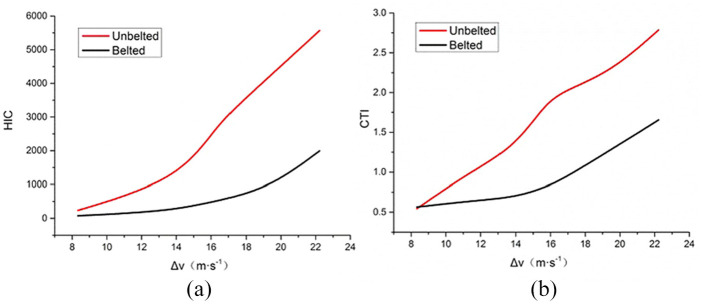
Head and chest simulation results when airbag deployment time is 20 ms: (a) HIC and (b) CTI.

Figure 3 displays the curve between head HIC, chest CTI, and delta-v when airbag deployment time was 20 ms. The result shows that there was a significant difference in HIC and CTI when the occupant was belted or not at the same airbag deployment time. The belted group had much lower values of injury than the unbelted group. Furthermore, with the increase of delta-v, the inter-group difference also increased. Under other airbag deployment time, a similar pattern was displayed.

**Figure 4.**
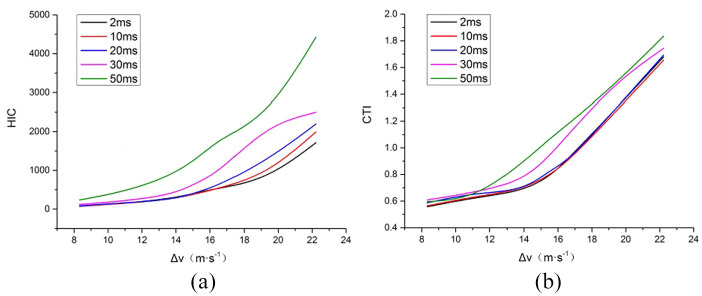
Head and chest simulation results under different airbag deployment times when belt was used: (a) HIC and (b) CTI.

Figure 4 shows the curve between head HIC, chest CTI, and delta-v at different airbag deployment times when the occupant was belted. The result shows that when the seatbelt was worn, the severity of occupant’s head and chest injuries increased along with the increase of the airbag deployment time. But the curves for 2, 10, and 20 ms were similar, which means that when the deployment time was less than 20 ms, the impact of deployment time on head and chest injuries was small. When the deployment time was greater than 20 ms, along with the increase of the time, both HIC and CTI had significant increases, but the increased amplitude of HIC was greater than CTI. This indicates that the deployment time had greater influence on the head injury than chest injury. Through analysis on Figures 3 and 4, it can be concluded that delta-v, seatbelt condition, and airbag deployment time have a great influence on the severity of occupant’s head and chest injuries.

### Neural network prediction results

**Figure 5.**
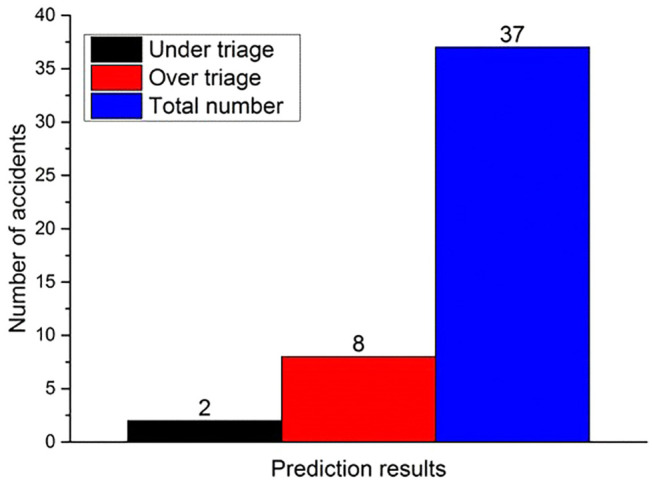
Neural network prediction results.

Based on the 37 cases of real-world traffic accidents with EDR data and injury information, the delta-v, seatbelt condition, and airbag deployment time were inputted into the neural network model for the prediction of head and chest injuries AIS codes. The predicted results were then compared with the actual injury severity. It was found that among the 37 cases, there was a consistency between predicted and actual injury results for 27 cases. And for eight cases, the predicted results were higher than the actual ones, while predicted results of two cases were lower than the actual injury severity (Figure 5).

### Model validation result

**Figure 6.**
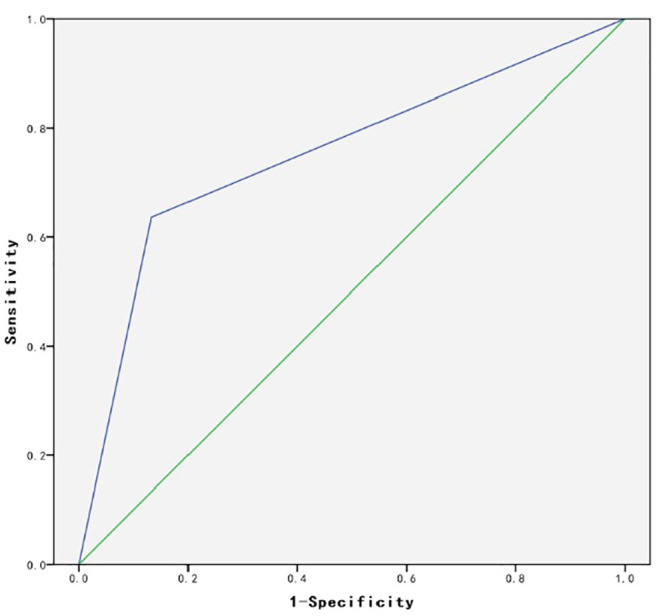
ROC analysis results.

Figure 6 illustrates the ROC analysis results, showing that AUC equals 0.747 (*p* = 0.011, *p* < 0.05). Thus, the AUC was greater than 0.7 but lower than 0.9, indicating a medium level of accuracy.

## Discussion

The validated FE and MADYMO models were used in this research to ensure the accuracy and reliability of the reconstructed results. The mixed-level orthogonal trial was adopted to simulate the situations under different collision speed, restraint system conditions, and airbag deployment time. Based on the statistical analysis on traffic accidents with EDR data, the airbags seldom deploy with collision speed under 30 km/h in real-world accidents. At collision speed of over 80 km/h, the death rate of occupants is so high that no study is necessary. Therefore, the collision speeds were set at seven levels from 30 to 80 km/h. The EDR data showed that the airbag deployment time hardly exceeded 60 ms, with the minimum value at 1 ms and average value at 22 ms. Thus, it was set at six levels from 2 to 50 ms. The simulation results showed that delta-v, seatbelt condition, and airbag deployment time were highly relevant to the injury severity. As delta-v increased, the head and chest injuries significantly increased. This means that delta-v was an important parameter affecting the severity of injury. The belted group was less severely injured than the unbelted group. As delta-v increased, the inter-group difference was greater. This indicates that when the intensity of collision increased, the protection capability of seatbelt was more significant. Fischer et al.^20^ and Gabauer and Gabler^21^ also studied the influence of seatbelt on injury severity. Their results showed that the unbelted group had a 42% higher AIS 3+ injury rate than the belted group. The airbag deployment time had influence on both head and chest injuries. The longer it took for the airbag to deploy, the greater was the injury to the head and chest. As shown in Figure 4, the airbag deployment time had a greater influence on the head injury than the chest injury.

The airbag is triggered by accelerometers in combination with sophisticated control logics. Subject to the type of collision, the deployment time varies from 10 to 30 ms after the first impact.^22^ In this study, the minimum airbag trigger time was 2 ms and the maximum could reach to about 60 ms, according to the EDR data of 37 real cases. Mismatched airbag trigger time may reduce the effectiveness of protection.^23^ The HIC and CTI values increase with increasing trigger time when the time was greater than 20 ms in this research. Tang et al.^24^ and Zhou et al.^25^ also found that an earlier airbag trigger time could reduce the injury risk of female drivers. However, Deng pointed out that injuries were associated with many factors, and there is an interaction between the airbag-head separation distance and the airbag trigger time. Therefore, in the future research, the body–airbag distance and body size should be taken as optimization factors.^26^

The value of delta-v was determined by the pre-crash speed and the rigidness of the crash target. However, the pre-crash speed was not a direct determinant parameter of the collision intensity. It was excluded from being an injury factor because the pre-crash speed could be high, but the collision intensity may not. In real-world cases, the pre-tensioning time of seatbelt is often consistent with the airbag deployment time. According to the theoretical analysis, the seatbelt pre-tensioning time should have similar influence on injuries to the airbag deployment time. But in this study, the groups were categorized as belted and unbelted. Therefore, this research did not consider pre-tensioning time of the seatbelt as a factor in the predicting model.

AACN assists in the fast EMS response after a traffic accident took place. Based on previous research, AACN can reduce the response time, which contributed to a death rate reduction by 1.5 to 20 percent.^27,28^ Urgency has a couple of versions, with the latest version being Urgency 2012. The additional algorithm also includes Onstar AACN, Toyota-Nihon University algorithm, and Honda-Nihon University “full model” algorithm. In summary, these algorithms are all based on statistical algorithm. However, the limitations of regression models must be realized. For instance, regression models described in the literature are typically linear models, which fail to capture the non-linear effects or interactions between the variables in the crash environment (e.g. effectiveness of wearing a seatbelt on injury outcome is dependent on the direction of the crash). Moreover, current statistical models lack details on crash information necessary for accurate and specific injury risk prediction. Finally, post-collision parameters collected from vehicle deformation, braking traces, and accident reconstruction may have poor accuracy. As key parameter of injury severity, delta-v is difficult to assess through accident investigation and reconstruction. But with the EDR data, the delta-v during collision is directly available. Although several studies show that the delta-v recorded in EDR has an error rate of about 3.5% to 6.6%,^29^ it is still a perfect data resource for injury prediction. The seatbelt wearing condition is 100% accurate from the EDR data. Although the injury prediction algorithm in this study is derived from computer simulation, real-world cases with EDR data are used to validate the established prediction algorithm for the purpose of better accuracy and reliability.

A binomial logistic regression is one of the most commonly used models to predict health-related outcomes.^30^ Some limitations of regression methods include considering a default distribution, such as the normal distribution for response variables, and the linearity of the proposed relationship. In practice, if the actual data do not have the assumed conditions, the use of these methods is not possible or is accompanied by a significant error.^31^ A neural network model is made up of layers of neurons, with the neurons in one layer connected to the neurons in the next. Each neuron is a computational unit that multiplies its input values by a corresponding set of learnable weight parameters, sums the multiplied values, transforms the summed value using a non-linear activation function, and outputs the transformed value.^32^ Using the learning methods for correcting itself, artificial neural network is not affected by interactions between factors, so it could provide much more accurate predictions.^33,34^ Besides, non-linear mapping between the factors and all levels of AIS could be established by artificial neural network, but the logistic regression only outputs binary predictions.

The established neural network model was used to predict injury severity based on the EDR data. The ROC result shows that the established algorithm presented medium-level prediction accuracy. Japanese researchers^35^ have validated the accuracy of Urgency Type C and Urgency 2012 by 135 real-world traffic accidents with AUC values of 0.751 and 0.838, respectively.^36^ Comparatively, the neural network model had similar accuracy to the Urgency Type C model but was not as accurate as the latest version of Urgency (Urgency 2012). Possible reason for this difference was that the model in this study did not consider the influence of age, gender, and vehicle type; eight cases with actual slight injuries were predicted with severe injury results, meaning the current prediction could sometimes be higher than the actual injury. Through analysis, the overpredicted cases typically showed higher level of delta-v, but severe injuries did not occur in those accidents. This difference mainly resulted from the vehicle-type variance, because different types of vehicles provide different occupant collision protection capabilities.

This study focused on the development and validation of a computational methodology for post-crash injury risk assessment. However, it was only a preliminary research; one of the major shortages was that the intrusion of the vehicle interior components was not simulated accurately due to lack of sufficient modeling details. Furthermore, the established model is only suitable for front impact condition and is not applicable for all real-world crash scenarios. Clearly, computational methodology has their relative merits as well as their inherent limitations. From the results derived in this study, it is speculated that computational methodology may have a greater potential for developing injury risk estimation algorithms suitable for AACN applications.

It is difficult to obtain the vehicle dynamic parameters through post-crash investigation. Anticipated advances in sensor technology involving occupant-adaptive restraints and stability control devices lead credence to the belief that the accuracy of information required for injury prediction will be significantly improved. The EDR records the static–dynamic parameters during collision, and these data can serve as an important supplement for injury prediction models. If the EDR data could be adopted in the investigation of in-depth traffic accidents, to replace the traditional method where some parameters are difficult to be obtained, it could be helpful in the optimization of injury models.

## Conclusion

The computer simulations, neural network, and EDR data were used to develop and validate the occupant injury prediction model, in which it was found that delta-v, seatbelt wearing condition, and airbag deployment time are important factors affecting occupant’s head and chest injuries. The validation result reveals that the established model has a medium-level prediction accuracy, almost similar to previous research. With the development of in-vehicle sensor technologies, the method derived in this study has a greater potential for developing injury risk estimation algorithms suitable for AACN applications, which could assist in medical decision-making and medical care.

## Appendix 1

1. Head injury risk function as a function of Head Injury Criterion (HIC)

(4)
$$P{\left(\mathrm{AIS}⩾1\right)}_{\mathrm{head}}=\frac{1}{1+e^{\left(1.54+\frac{200}{\mathrm{HIC}}\right)-0.0065\mathrm{HIC}}}$$

(5)
$$P{\left(\mathrm{AIS}⩾2\right)}_{\mathrm{head}}=\frac{1}{1+e^{\left(2.49+\frac{200}{\mathrm{HIC}}\right)-0.0048\mathrm{HIC}}}$$

(6)
$$P{\left(\mathrm{AIS}⩾3\right)}_{\mathrm{head}}=\frac{1}{1+e^{\left(3.39+\frac{200}{\mathrm{HIC}}\right)-0.0037\mathrm{HIC}}}$$

(7)
$$P{\left(\mathrm{AIS}⩾4\right)}_{\mathrm{head}}=\frac{1}{1+e^{\left(4.90+\frac{200}{\mathrm{HIC}}\right)-0.0035\mathrm{HIC}}}$$

(8)
$$P{\left(\mathrm{AIS}⩾5\right)}_{\mathrm{head}}=\frac{1}{1+e^{\left(7.82+\frac{200}{\mathrm{HIC}}\right)-0.0043\mathrm{HIC}}}$$

(9)
$$P{\left(\mathrm{AIS}⩾6\right)}_{\mathrm{head}}=\frac{1}{1+e^{\left(12.24+\frac{200}{\mathrm{HIC}}\right)-0.0057\mathrm{HIC}}}$$

2. Chest injury risk function as a function of Combined Thoracic Index (CTI)

(10)
$$P{\left(\mathrm{AIS}⩾2\right)}_{\mathrm{chest}}=\frac{1}{1+e^{4.847-6.036\mathrm{CTI}}}$$

(11)
$$P{\left(\mathrm{AIS}⩾3\right)}_{\mathrm{chest}}=\frac{1}{1+e^{8.224-7.125\mathrm{CTI}}}$$

(12)
$$P{\left(\mathrm{AIS}⩾4\right)}_{\mathrm{chest}}=\frac{1}{1+e^{9.872-7.125\mathrm{CTI}}}$$

(13)
$$P{\left(\mathrm{AIS}⩾5\right)}_{\mathrm{chest}}=\frac{1}{1+e^{14.242-6.589\mathrm{CTI}}}$$
